# The third intracellular loop of *Drosophila* Lilipod is required for protein function *in vivo* and can mediate protein-protein interactions *in vitro*

**DOI:** 10.1371/journal.pone.0325326

**Published:** 2025-06-04

**Authors:** Merin Vellooparambil Roy, Scott J. Neal, Francesca Pignoni

**Affiliations:** 1 Department of Biochemistry and Molecular Biology, Upstate Medical University, Syracuse, New York, United States of America; 2 Department of Neuroscience and Physiology, Upstate Medical University, Syracuse, New York, United States of America; Kookmin University, KOREA, REPUBLIC OF

## Abstract

The evolutionarily conserved Lipocalin-Interacting Membrane Receptor (LIMR) family (InterPro: IPR006876) consists of transmembrane (TM) proteins characterized by 9 TM domains (TMDs). Their reported biological functions are diverse and remain poorly understood. In previous work, we showed that the fly family member Lilipod (Lili) impacts biological processes regulated by the fly BMP/TGF-β ligand Decapentaplegic (Dpp), including germline stem cell self-renewal in the *Drosophila* ovary, dorsal closure during embryonic development and wing vein formation at the pupal stage. Based on this genetic evidence, Lili directly or indirectly enhances bone morphogenetic protein (BMP) signaling. In the ovary, Lili functions between the activated type I BMP receptor and the SMAD intracellular transducer. To gain insight into Lili function at the cellular and molecular levels, we probed the functional significance of its largest intracellular loop, Intracellular Loop 3 (ICL3). Through mutational analysis, we mapped sequences critical for Lili function *in vivo* to the evolutionarily conserved regions of ICL3. Additionally, we showed that fly-human chimeric proteins in which Lili ICL3 is replaced with the ICL3 of its human homologs, LMBR1 and LMBR1L, can rescue *lili* null-mutant phenotypes. Using ICL3 as bait in an unbiased Yeast 2-Hybrid (Y2H) screen, we identified putative interactors, including the BMP signaling cascade components Mad, Sara, Nup93 and Nup358, and further Y2H analyses identified distinct regions on ICL3 as potentially important for protein binding. Taken together, our work has identified ICL3 as a region that is critical for Lili protein function, most likely via its mediation of protein-protein interactions (PPIs).

## Introduction

In humans, the integral membrane proteins LMBR1 and LMBR1L (LMBR1-like; formerly LIMR – Lipocalin Interacting Membrane Receptor) share significant sequence homology and predicted transmembrane topology. Although a number of findings suggest diverse and possibly tissue specific functions for these proteins, their biological roles are still poorly understood. When first identified, LMBR1L was shown to bind and mediate cellular endocytosis in cell culture of human tear lipocalin LCN1 [1–3] – a carrier of lipophilic ligands [4] – as well as other carrier proteins, such as the bovine lipocalin β-lactoglobulin [5] and uteroglobin [6]. However, the variable strength of these interactions has raised questions about their physiological significance [1].

Over the last decade, LMBR1L has been shown to bind the Androgen Receptor associated protein MAGEA11 *in* vitro [7] and the aryl hydrocarbon receptor repressor AHRR in monocytic THP-1 cells to promote NF-κB mediated inflammation [8]. Lastly, two recent studies strongly implicate LMBR1L in lymphopoiesis and retinal angiogenesis where it negatively regulates Wnt and Norrin signaling by stabilizing the destruction complex, regulating cascade components’ maturation or expression, and promoting ER-associated degradation (ERAD) of signaling components [9,10]. Less is known of LMBR1. Two very recent papers report that LMBR1 suppression, via miR-758 or PAR1 activation, is critical for BMP2/4 activation during osteogenic differentiation of periodontal ligament stem cells [11,12]. These reports leave us with a complex picture of this protein family with little clarity as to their biological function.

**Fig 1:**
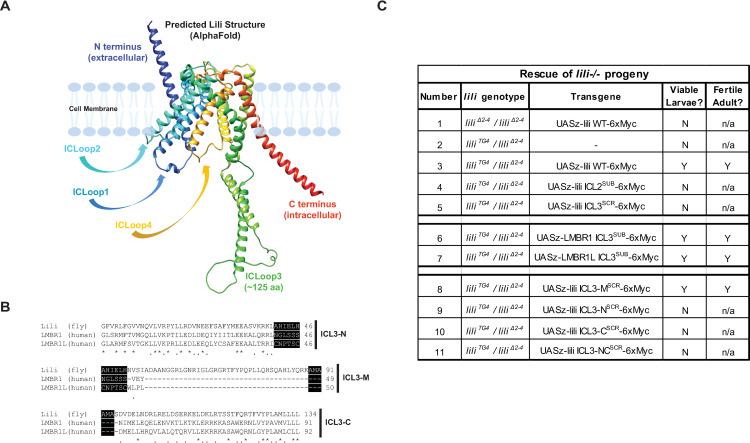
Mutational analysis of Lili ICL3. A) Lili structure as predicted in the AlphaFold Database (AFDB accession AF-Q9VC35-F1). Alternate *de novo* structure prediction using Robetta (https://robetta.bakerlab.org) returned similar results (not shown). In all cases, confidence in the predictions was highest in the 9 presumptive TMDs, moderate in loops (excluding the low confidence ICL3-M region) and intermediate or low, approaching the N- and C-termini of the protein. B) Sequence alignment of ICL3 regions of *Drosophila* Lili and of its human homologs LMBR1 and LMBR1L, broken down into 3 sub-fragments (ICL3-N, ICL3-M and ICL3-C). Alignment scores appear below the sequences (“*” identical residues, “.” similar residues). Residues highlighted in black represent regions of overlap between adjacent fragments. Note that the middle region (ICL3-M) is absent from LMBR1 and LMBR1L. C) Summary of *in vivo* rescue experiments by Lili WT and modified variants; X^SUB^ and X^SCR^ denote substitution or scrambling of amino acid sequence X, respectively (see Methods for scrambling). *lili* null mutants *lili*^*Δ2*^^*–4*^/ *lili*^*Δ2*^^*–4*^ (row 1) and *lili*^*TG4*^/ *lili*^*Δ2*^^*–4*^ (rows 2-11) are larval lethal, unless rescued by a combination of the GAL4 driver and the *UASz-Lili* transgene, as indicated. WT Lili can rescue *lili*^*TG4*^/ *lili*^*Δ2*^^*–4*^ to the adult stage (row 3). When the ICL3 region is substituted with ICL2 sequence (row 4) or scrambled (row 5), the larval lethality of *lili*^*TG4*^/ *lili*^*Δ2*^^*–4*^ is not rescued. Substitution of Lili ICL3 with the LMBR1 ICL3 (row 6) or LMBR1L ICL3 (row 7) rescues *lili*^*TG4*^/ *lili*^*Δ2*^^*–4*^ lethality. Scrambling of the middle region (row 8), but not the ICL3-N (row 9 and 11) or ICL3-C (row 10 and 11) regions, also rescues *lili*^*TG4*^/ *lili*^*Δ2*^^*–4*^. In all cases, rescued females are fertile; *: *p* < 0.001. Observations supporting this summary data are provided in S1 Table.

An underutilized approach in the investigation of these proteins lies in leveraging simpler, genetically amenable animal models. In *Drosophila*, the protein Lilipod (Lili) is the only homolog of LMBR1 and LMBR1L. Like its human counterparts, Lili comprises a single, large LMBR1 domain (pfam04791) that spans all 9 TMDs (Fig 1A). All three proteins share a noteworthy feature in the presence of a large intracellular loop, the third loop (ICL3), which is much larger than any of the other intracellular or extracellular loops (Fig 1A).

We previously showed that Lili promotes signaling by the TGF-β ligand Dpp (the fly BMP2/4 homolog) in three Dpp-regulated processes: the maintenance of ovarian germline stem cells (GSC), vein development in the wing, and dorsal closure in the *Drosophila* embryo [13]. Genetic evidence places Lili’s role intracellularly [13], between the type 1 BMP receptor Thickveins (Tkv) and the intracellular SMAD transducer Mothers against Dpp (Mad) in GSCs. However, the role of Lili at a cellular or molecular level is still unknown. Owing to its length and mostly polar constitution (S1A Fig), ICL3 offers a hydrophilic surface that appears accessible to intracellular factors. Considering this prominent structural feature of Lili and its potentiation of signaling downstream of the Dpp receptor, we sought to investigate the requirement for ICL3 in Lili function and its potential role in mediating protein-protein interactions (PPIs).

Here, we show that the lethality and sterility of *lili* null-mutants can be rescued by exogenous wild-type (WT) Lili as well as Lili-LMBR1 or Lili-LMBR1L chimeric *Drosophila* proteins containing human ICL3 loops. Conversely, Lili variants lacking a wild type ICL3 region, via deletion or modification, cannot rescue the *Drosophila lili* null mutant to viability. Through a Lili ICL3-based Y2H screen, we have identified 144 putative interactors, including several proteins implicated in Dpp signaling. These findings suggest an essential role for ICL3 in the function of Lili *in vivo*, with a possible role in mediating PPIs. Lastly, we found that the N- and C-terminal portions of the loop are functionally required for phenotypic rescue *in vivo* and for protein interactions *in vitro*.

## Results

### ICL3 is required for Lili function

Lili, like its human homologs LMBR1 and LMBR1L, consists of an extracellular N terminus, an intracellular C tail and 9 TM domains connected by four extracellular and four intracellular loops. The third intracellular loop, ICL3, spans ~125 amino acids (aa) (Fig 1A), whereas the other loops contain just 18–31 aa. All loops consist mostly of polar residues (S1A Fig).

Sequence alignment of ICL3 with protein family members from other species (Clustal Omega Multiple Sequence Alignment) highlights its significant evolutionary conservation with distinct regions that differ in the extent of sequence homology (S1B Fig). The proximal N and distal C regions of Lili ICL3 share 55% and 44% sequence similarity, respectively, with human LMBR1 and LMBR1L (Fig 1B). Surprisingly, the N and C regions are separated by a middle (M) segment that is completely absent in the human proteins (Fig 1B, S1B Fig). Hence, for the purpose of our study, we considered the loop to consist of three different regions, named ICL3-N, ICL3-M, and ICL3-C (Fig 1B). Notably, the conserved ICL3-N and ICL3-C sections are predicted to each have helical structure and to come together to form a stalk that projects intracellularly, whereas ICL3-M constitutes an unstructured connection between the two helical regions (Fig 1A).

To test whether ICL3 is important for Lili function *in vivo*, we first developed a phenotypic rescue assay to assess the ability of WT and modified Lili proteins to rescue the lethality of *lili* null-mutant progeny and adult female sterility. While viability through the larval stage does not require rescue of dorsal closure in embryogenesis (due to maternal Lili), female fertility requires rescue of GSC maintenance in the ovary [13].

We previously showed that homozygous *lili* null mutants die during the 1^st^ or 2^nd^ larval stage (L1 or L2) but can be partially rescued to viability by expression of WT protein (*UASt-lili WT*) in the soma, broadly and at a low level [13]. However, rescue of sterility was not possible due to inefficient expression of the *UASt* transgene in the germline. To develop a robust rescue assay, we expressed exogenous Lili in homozygous mutant backgrounds in the pattern of the endogenous *lili* gene, in both soma and germline. We achieved this by using the *lili*^*TG4*^ allele in combination with *UASz-lili* transgene. *lili*^*TG4*^ is both a null mutant allele and a GAL4 driver, generated by the insertion of a ‘Trojan’ exon encoding the GAL4 protein within the first intron of the *lili* gene [14] (S2 Fig). This insertion leads to (a) the expression of GAL4 under the control of the *lili* gene regulatory DNA and (b) the creation of a *lili* null allele due to the termination of transcription within the Trojan exon, thereby causing a truncation early in the *lili* gene ORF (S2 Fig).

To validate our assay, we tested whether null mutant progeny could be rescued to fertile female adults by a *UASz-lili WT* transgene. Combining the *lili*^*TG4*^ GAL4-expressing allele with the null allele *lili*^*Δ2*^^*–4*^ (S2 Fig, new CRISPR allele; see Methods) and a *UASz-lili WT-6xMyc* transgene restored viability and female fertility (Fig1C row 3; S1 Table).

Next, to address whether ICL3 is important for Lili function, we repeated this test while expressing Lili mutant variants (*UASz-lili ‘variant’-6xMyc* transgenes). To begin, we either substituted ICL3 with the sequence of second intracellular loop (ICL2; *UASz-lili ICL2*^*SUB*^*-6xMyc*) or scrambled the entire ICL3 aa sequence (see Methods; *UASz-lili ICL3*^*SCR*^*-6xMyc*). Protein structure prediction was used to ensure that overall protein topology was not grossly affected by these sequence substitutions. In neither case did we observe rescue of *lili*^*TG4*^/*lili*^*Δ2–4*^ lethality (Fig 1C rows 4–5; S1 Table). Failure to rescue was not due to a lack of protein expression, as shown in S2 cells and in stage 9–10 egg chambers (S3A-D Fig).

Taken together, these data suggest a functional role for Lili ICL3.

### ICL3 of human homologs can substitute for Lili ICL3

Lili and its human homologs share 50.7% sequence similarity in ICL3, when excluding the M region (Fig 1B). Given the conservation, we wanted to know if these related ICL3s from human proteins could functionally replace Lili ICL3. Hence, we substituted Lili ICL3 with the corresponding ICL3 sequence of either LMBR1 (Fig 1C row 6) or LMBR1L (Fig 1C row 7), generating the transgenes *UASz-lili LMBR1 ICL3*^*SUB*^*-6xMyc* and *UASz-lili LMBR1L ICL3*^*SUB*^*-6xMyc.* These fly-human chimeric proteins were robustly expressed *in vitro* and *in vivo* (S3B-C Fig). Strikingly, both successfully rescued *lili*^*TG4*^/*lili*^*Δ2*^^*–4*^ larval lethality, producing apparently normal flies and fertile adult females (Fig 1C rows 6–7; S1 Table).

These findings demonstrate that the human LMBR1/LMBR1L ICL3s can fulfill critical functions of Lili ICL3 *in vivo*.

### The conserved N and C regions of ICL3, but not the unique M region, are necessary for Lili function *in vivo*

As mentioned above, the ICL3-M region is absent in LMBR1 and LMBR1L. Their ability to rescue *lili^TG4^*/*lili^Δ2^^–4^* lethality and female fertility, despite the absence of ICL3-M, suggested that the middle region of the loop is not essential for Lili function. To directly test this hypothesis, we scrambled the residues of ICL3-M, leaving the N and C regions unchanged and confirmed that the resulting Lili variant was robustly expressed *in vitro* and *in vivo* (S3B-C Fig). Consistent with our hypothesis, we found that *UASz-lili ICL3-M^SCR^*-*6xMyc* robustly rescued larval lethality and female fertility of *lili^TG4^*/*lili^Δ2^^–4^* flies (Fig 1C row 8; S1 Table). This finding confirms that the middle region of ICL3 is not required for Lili function in these processes.

Next, we tested whether either or both conserved regions of ICL3 are required for Lili function *in vivo*. To this end, we scrambled the residues of either or both ICL3-N and ICL3-C to generate the transgenes *UASz-lili ICL3-N^SCR^*-*6xMyc*, *UASz-lili ICL3-C^SCR^*-*6xMyc* and *UASz-lili ICL3-NC^SCR^*-*6xMyc*. We confirmed that all three Lili variants were robustly expressed *in vitr*o (S3A Fig, S3C Fig) and *in vivo* (S3C-D Fig). In fact, expression of these mutants was comparable to that of *UASz-lili WT* in S2 cells (S3A Fig, S3C Fig) and in stage 9/10 egg chambers (S3C-D Fig). Then, we tested these variants’ ability to rescue *lili^TG4^*/*lili^Δ2^^–4^* larval lethality and, if the latter was rescued, female fertility. Interestingly, disrupting the sequence of either conserved region (Fig 1C, rows 9–10; S1 Table) resulted in a failure to rescue lethality; the doubly scrambled variant was also unable to rescue (Fig 1C, row 11; S1 Table).

Altogether, these data strongly suggest that the non-conserved M region is dispensable for Lili function whereas the evolutionarily conserved N and C regions are both essential.

### Identifying putative interacting partners of Lili using an ICL3-based Y2H screen

Given that ICL3 appears to be (1) required for Lili function *in vivo*, (2) exposed to the intracellular space, and (3) devoid of any characterized functional domains, we hypothesized that Lili’s ICL3 might be responsible for mediating key protein-protein interactions.

**Fig 2:**
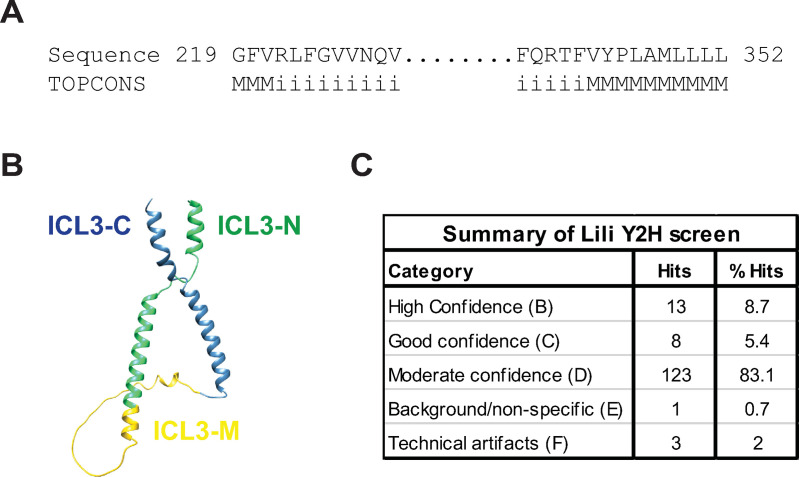
Identification of putative ICL3 binding proteins using a Y2H screen. A) Consensus prediction of transmembrane protein topology of selected 134 aa-long Lili ICL3 region using TOPCONS. “M” stands for transmembrane residues and “i” for intracellular loop residues. B) Structure of the selected Lili ICL3 bait as predicted by AlphaFold2. It is similar to the loop’s structure in the context of the full-length protein (see Fig 1A). C) Summary results of Y2H screen using the 134 aa Lili ICL3 as bait, screened against the *Drosophila* Embryo_RP2 prey library. A total of 148 unique interactors were identified (see Table 1).

To explore this possibility, we employed an unbiased Y2H approach. We selected a 134 aa fragment (Fig 2A) to be used as bait, relying on TOPCONS to generate a consensus prediction for the transmembrane topology of Lili. This fragment showed a similar predicted 3D structure as the ICL3 in full-length Lili (Fig 2B). Thereafter, the ICL3-containing bait (ICL3) was screened against the *Drosophila* Whole Embryo_RP2 prey library (Hybrigenics). The unbiased screen resulted in the detection of 275 interactions with 148 unique proteins, out of which 144 were putative interactors of ‘high to moderate’ confidence (Fig 2C, Table 1, S1 File); 4 were categorized as technical artifacts or non-specific interactions.

**Table 1:** Identified ICL3-binding preys.

Gene^1^	PBS^2^	#^3^	Notes^4^
Aats-lys	B	3	Lysyl-tRNA synthetase, transcript variant A
CG17162	B	6	CG17162, transcript variant C
CSN5	B	6	COP9 complex homolog subunit 5, transcript variant A
Clect27	B	5	C-type lectin 27kD, transcript variant A
Egfr	B	5	Epidermal growth factor receptor, transcript variant B
Gbeta13F	B	3	G protein beta-subunit 13F, transcript variant C
Irp-1B	B	5	Iron regulatory protein 1B
Nc73EF	B	3	Neural conserved at 73EF, transcript variant A
**Sesn** ^5^	**B**	**4**	**Sestrin**, transcript variant A
Tsf2	B	4	Transferrin 2
sina	B	3	Seven in absentia, transcript variant A
swm	B	5	Second mitotic wave missing, transcript variant A
und	B	3	Uninitiated, transcript variant A
CDase	C	2	Ceramidase, transcript variant A
CG14476	C	2	CG14476, transcript variant A
CG30497	C	2	CG30497, transcript variant A
CG6726	C	2	CG6726
**EDTP** ^**5**^	**C**	**2**	**Egg-derived tyrosine phosphatase**, transcript variant C
EF2	C	2	Elongation factor 2, transcript variant C
Grip	C	2	Glutamate receptor binding protein, transcript variant B
Men	C	2	Malic enzyme, transcript variant A
Abl	D	1	Abl tyrosine kinase, transcript variant C
AhcyL1	D	1	Adenosylhomocysteinase like 1
Alp1	D	1	Alkaline phosphatase 1, transcript variant A
alpha-Man-Ia	D	1	Alpha-Mannosidase class I a, transcript variant O
Argk	D	1	Arginine kinase, transcript variant A
Asap	D	1	ArfGAP with SH3 domain, ankyrin repeat and PH domain, transcript variant B
Atg2	D	2	Autophagy-related 2
bel	D	1	Belle, transcript variant B
betaTub56D	D	1	Beta-Tubulin at 56D, transcript variant A
betaTub60D	D	1	Beta-Tubulin at 60D, transcript variant B
bin3	D	1	Bicoid-interacting protein 3, transcript variant D
brun	D	1	Brunelleschi
bt	D	1	Bent, transcript variant C
Btk29A	D	1	Btk family kinase at 29A, transcript variant A
ca	D	1	Claret, transcript variant A
cana	D	2	CENP-ana, transcript variant A
CG10077	D	1	CG10077, transcript variant A
CG16974	D	1	CG16974, transcript variant A
CG17018	D	1	CG17018, transcript variant F
CG17065	D	1	CG17065, transcript variant B
CG17109	D	1	CG17109, transcript variant B
CG17597	D	1	CG17597, transcript variant B
CG17672	D	1	CG17672
CG17896	D	1	CG17896, transcript variant A
Cg25C	D	1	Collagen type IV, transcript variant A
CG2915	D	1	CG2915, transcript variant A
CG31004	D	2	CG31004, transcript variant C
CG3108	D	1	CG3108
CG31140	D	1	CG31140, transcript variant A
CG32549	D	1	CG32549, transcript variant E
CG32564	D	1	CG32564
CG3295	D	1	CG3295
CG33331	D	1	CG33331
			
CG3434	D	1	CG3434
CG34422	D	1	CG34422, transcript variant C
CG3808	D	1	CG3808
CG43795	D	1	CG43795
CG4538	D	1	CG4538, transcript variant C
CG4670	D	1	CG4670, transcript variant B
CG4752	D	1	CG4752
CG5484	D	1	CG5484, transcript variant A
CG6204	D	1	CG6204
CG7896	D	1	CG7896
CG8907	D	1	CG8907, transcript variant A
CG9086	D	1	CG9086, transcript variant B
CG9509	D	1	CG9509, transcript variant B
Chc	D	1	Clathrin heavy chain, transcript variant G
Clamp	D	1	Chromatin-linked adaptor for MSL proteins, transcript variant B
cno	D	1	Canoe (cno), transcript variant C
cno	D	1	Canoe (cno), transcript variant D
Cont	D	1	Contactin
Ctr9	D	1	Ctr9, transcript variant A
CtsB1	D	1	Cathepsin B1
CycE	D	1	Cyclin E, transcript variant B
disco-r	D	1	Disco-related, transcript variant A
dp-varI	D	1	Dumpy, transcript variant I
eIF3m	D	1	Eukaryotic translation initiation factor 3 subunit m, transcript variant A
Eno	D	1	Enolase, transcript variant F
eve	D	1	Even skipped
Exo70	D	1	Exocyst 70
Faa	D	1	Fumarylacetoacetase
FASN1	D	1	Fatty acid synthase 1, transcript variant C
Fer1HCH	D	1	Ferritin 1 heavy chain homologue, transcript variant A
GatA	D	1	Glutamyl-tRNA amidotransferase subunit A
Gfat2	D	1	Glutamine:fructose-6-phosphate aminotransferase 2
GS	D	1	Glutathione synthetase, transcript variant E
GstD1	D	1	Glutathione S transferase D1, transcript variant B
Hsp83	D	1	Heat shock protein 83, transcript variant B
Idgf6	D	1	Imaginal disc growth factor 6, transcript variant B
Iml1	D	1	Increased minichromosome loss 1, transcript variant E
Imp	D	1	IGF-II mRNA-binding protein, transcript variant A
Inr-a	D	1	Inverse regulator a, transcript variant D
Jon99Ciii	D	1	Jonah 99Ciii, transcript variant B
Kap3	D	1	Kinesin associated protein 3, transcript variant F
l(1)G0334	D	2	Lethal (1) G0334, transcript variant A
l(3)72Ab	D	1	Lethal (3) 72Ab
***Mad*** ^5^ ^,^ ^6^	***D***	***1***	***Mothers against dpp*** *, transcript variant B*
Mat89Ba	D	1	Maternal transcript 89Ba
Mcr	D	1	Macroglobulin complement-related
mRpS22	D	1	Mitochondrial ribosomal protein S22
Nlg2	D	1	Neuroligin 2, transcript variant C
noi	D	1	Noisette
Not1	D	1	Not1, transcript variant D
Nrt	D	1	Neurotactin, transcript variant A
*Nup358*	*D*	*1*	*Nucleoporin 358kD*
*Nup93−1*	*D*	*1*	*Nucleoporin 93kD-1*
			
pes	D	1	Peste, transcript variant H
Plc21C	D	1	Phospholipase C at 21C, transcript variant C
Plod	D	1	Procollagen lysyl hydroxylase, transcript variant B
Pp2A-29B	D	1	Protein phosphatase 2A at 29B, transcript variant D
Pp2B-14D	D	1	Protein phosphatase 2B at 14D, transcript variant C
PPO1	D	1	Prophenoloxidase 1
Ptip	D	1	PAX transcription activation domain interacting protein
pum	D	1	Pumilio, transcript variant A
pzg	D	1	Putzig, transcript variant A
r	D	1	Rudimentary, transcript variant A
rib	D	1	Ribbon, transcript variant A
rno	D	1	Rhinoceros, transcript variant C
RpL18A	D	1	Ribosomal protein L18A
***Sara*** ^5^ ^,^ ^6^	***D***	***1***	***Smad anchor for receptor activation***
sca	D	1	Scabrous, transcript variant B
scramb2	D	1	Scramblase 2
Sec10	D	1	Secretory 10
Sec16	D	1	Secretory 16, transcript variant F
shot	D	1	Short stop, transcript variant A
sli	D	2	Slit, transcript variant C
smid	D	1	Smallminded, transcript variant A
SP1029	D	1	SP1029, transcript variant E
spen	D	1	Split ends, transcript variant A
spz4	D	1	Spatzle 4
Su(var)2–10 varD	D	1	Suppressor of variegation 2–10, transcript variant D
Synj	D	1	Synaptojanin, transcript variant B
Ten-a	D	1	Tenascin accessory, transcript variant E
toe	D	1	Twin of eyg, transcript variant A
trol	D	2	Terribly reduced optic lobes, transcript variant BA
Tsf3	D	1	Transferrin 3
unc-104	D	1	Uncoordinated-104, transcript variant E
UQCR-C1	D	1	Ubiquinol-cytochrome c reductase core protein 1, transcript variant B
Vha55	D	1	Vacuolar H[+]-ATPase 55kD B subunit, transcript variant A
vkg	D	1	Viking, transcript variant A
Wee1	D	1	Wee1 kinase, transcript variant B
Zw10	D	1	Zeste-white 10
Unknown	D	1	Homolog of GenMatch (Drosophila melanogaster); [https://www.ncbi.nlm.nih.gov/nuccore/CP020167.1- Droso – hom. of GenMatch]

^1^Gene symbol best matching aligned sequence.

^2^Computed global confidence score.

^3^Number of independent clones observed.

^4^Gene name and transcript information.

^5^Interactors selected for further study (**bold**).

^6^Selected interactors associated with Dpp signaling (***bold italics***).

Of the 144 putative interactors, four proteins – Mad, Sara, Nup93-1 and Nup358 – are known to play roles in Dpp/BMP signaling in *Drosophila*. Mad belongs to the SMAD family of transcription factors, which are phosphorylated when BMP receptors are activated following ligand binding [15,16]. Phosphorylated Mad can translocate to the nucleus where it turns on/off BMP-responsive genes [17]. *Drosophila* Sara (SMAD anchor for receptor activation) has been shown to recruit Protein Phosphatase 1C to Tkv, the BMP type 1 receptor. Thus, Sara negatively regulates BMP signaling by promoting receptor inactivation via dephosphorylation [18]. Nucleoporins Nup93 and Nup358 are components of the nuclear pore complex. In *Drosophila* cell culture, Nup93-1 and Nup358 were shown to promote pMad nuclear import by localizing the importin Moleskin to the nuclear periphery [19].

Other putative interactors of interest included two factors linked to mutant ovarian phenotypes. Sestrin (Sesn) and Egg derived Tyrosine Phosphatase (EDTP) function in the fly ovary and their loss-of-function leads to ovarian defects that result in reduced fecundity [20,21]. Sesn (Category B) and EDTP (Category C) fell into higher confidence categories than Mad, Sara, Nup93-1 and Nup358 (Table 1).

### Conserved regions of human and fly ICL3 interact with selected factors in Y2H assays

**Fig 3:**
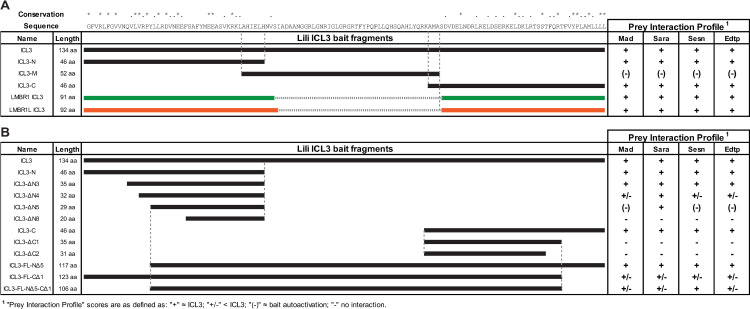
Analysis of human and fly ICL3-mediated PPIs using Y2H assays. A) Summary of Y2H interactions between ICL3, its fragments and selected prey; horizontal bars depict the regions of ICL3 provided as bait in the assay. Sequence conservation among fly and human proteins is reported above the primary sequence (“*” identical residues; “.” similar residues). Lili ICL3-Mad/Sara/Sesn/EDTP interactions obtained from the screen were consistent in pairwise Y2H assays. ICL3-N and ICL3-C fragments, but not ICL3-M, interacted with similar strength as Lili ICL3 with all prey tested. ICL3 of human LMBR1 and LMBR1L interacted with all prey tested, at similar strengths as Lili ICL3. Note that the middle region of ICL3 is absent in the human homologs. B) Summary of Y2H interactions between truncated ICL3 fragments and selected prey.

Among the 6 interactors mentioned above, we selected Mad, Sara, Sesn and EDTP for secondary testing by Hybrigenics (S4A-B Fig) and in our lab (Fig 3A) to confirm their interactions with Lili ICL3. All 4 preys consistently interacted with the Lili ICL3 bait, albeit with some variation in PPI strength (Fig 3A, S4 Fig, S2 File).

Next, we used Y2H assays to test whether subregions of the ICL3, already found to be required for Lili function *in vivo,* were also specifically needed to mediate these PPIs. For this, we generated different baits based on sequence conservation in ICL3; these included three slightly overlapping baits spanning the conserved N-terminal (ICL3-N; 46 aa) and C-terminal (ICL3-C; 46 aa) regions and the non-conserved middle region (ICL3-M; 52 aa); see Fig 1B. We tested whether these shorter fragments could independently interact with Mad, Sara, EDTP and Sesn. The ICL3-N and ICL3-C mimicked the full ICL3 bait in terms of strength of interaction with all prey (Fig 3A). On the contrary, the ICL3-M bait with empty prey vector (negative control) grew on -Leu/Trp/His (DO-3) selection media without and with 3-aminotriazole (up to 2 mM), suggesting significant bait autoactivation (S2 File). Growth on selective media was not enhanced by inclusion of Mad, Sara, EDTP or Sesn with ICL3-M (Fig 3A, S2 File). These findings indicate that both the conserved N and C regions of ICL3 can independently interact with each of the 4 selected putative partners and that the non-conserved ICL3-M region does not appear to specifically promote any of the four PPIs.

Moreover, we found that baits containing the human LMBR1 or LMBR1L ICL3 regions also interacted with *Drosophila* Mad, Sara, EDTP and Sesn, similarly to the fly ICL3 bait (Fig 3A). These findings support the notion that the ICL3-M region of Lili does not play a key role in the selected PPIs, as the human homologs naturally lack the M region. Additionally, the ability of LMBR1 and LMBR1L ICL3 to mediate PPIs with the fly prey proteins is consistent with their ability to rescue viability of *lili* null mutants (Fig 1C rows 6–7).

Taken together, these findings strongly suggest an important role for the conserved N and C regions of the ICL3 in Lili function, possibly via mediation of functional PPIs.

### Analysis of ICL3-N and ICL3-C mediated PPIs

To better understand the N and C regions of ICL3 as potential PPI domains, we generated fragments of ICL3-N and ICL3-C, progressively deleted from the fragments’ ends corresponding to the N-terminal and C-terminal base of the loop (Fig 3B). Progressive truncations of the N fragment first partially (ICL3-NΔ4) and then completely (ICL3-NΔ5) compromised the interactions with Mad, EDTP and Sesn. Yet, the same baits (ICL3-NΔ4 and ICL3-NΔ5) still robustly interacted with Sara. Further truncations resulted in the eventual loss of the interaction with Sara (ICL3-NΔ8), requiring the removal of over 50% of the N fragment (Fig 3B, S2 File). These findings suggest that there might be subtle differences in how Lili binds to its putative partners. In the C region, the deletion of 11 residues (ICL3-CΔ1) resulted in the loss of all interactions tested (Fig 3B, S2 File), suggesting that the deleted region is directly or indirectly critical to all 4 PPIs.

Next, we made a series of baits in which the regions deleted in ICL3-NΔ5 and ICL3-CΔ1 were removed singly or together from an otherwise full-length ICL3 fragment. Removal of the NΔ5 region alone resulted in no obvious weakening of the 4 PPIs as compared to ICL3 control (ICL3-FL-NΔ5, Fig 3B). Removal of the CΔ1 region alone in the context of ICL3 yielded a partial loss of interaction with all 4 putative partners (ICL3-FL-CΔ1, Fig 3B). Surprisingly, removal of both regions, NΔ5 and CΔ1, did not detectably further impair the 4 PPIs, over the loss of CΔ1 alone (ICL3-FL-NΔ5-CΔ1, Fig 3B). The latter findings may reflect insufficient sensitivity of the Y2H assay in detecting changes in PPI strength or complex contributions of these regions to specific PPIs that depend on additional residues that are not properly assessed by the selected baits.

## Conclusions

In this study, we show that the ICL3 of Lili is likely essential for its biological function and demonstrate its potential role in mediating PPIs.

Our *in vivo* mutational studies demonstrate, for the first time, the importance of ICL3 in Lili function. Interestingly, exome sequencing from 16 individuals affected by incomplete virilization has identified two missense mutations in the ICL3-C region of human LMBR1L [22]. Though preliminary, this finding also supports a potential role for ICL3 in LMBR1L protein function.

Deletion analysis of ICL3 binding to selected prey (Mad, Sara, Sesn and EDTP) suggests that the evolutionarily conserved N and C regions play a significant role in mediating these PPIs. A simple explanation for the participation of both regions in binding to the same prey is offered by the predicted tertiary structure of Lili, in which the N and C regions of ICL3 are closely apposed (Fig 1A). Moreover, a functional role for the conserved N and C regions of ICL3 is also supported by the ability of the human LMBR1/LMBR1L ICL3 fragments to substitute for Lili ICL3 in the fly protein *in vivo* (phenotypic rescue) and *in vitro* (Y2H) – despite the lack of the ICL3-M region found in the fly but not the human proteins. However, our deletion analysis was of limited usefulness in identifying aa central to specific PPIs. We believe that a better approach lies in mutational scanning of the ICL3 first in yeast, followed by PPI testing by immunoprecipitation in the context of the full-length Lili protein.

Our unbiased Y2H screen identified several BMP signaling components as potential partners of Lili as well as a plethora of other factors with diverse functions. If Lili’s role in Dpp signaling occurs via Mad, Sara, and/or Nup93/Nup358, Lili may function by enhancing Mad phosphorylation via negative regulation of Sara or by promoting Nup93/Nup358-assisted pMad nuclear import. An assessment of the many other interactors requires additional work. Among these putative interactors, GO analyses did not reveal any predominant biological process or molecular function. We are disappointed but not surprised by this outcome, which we believe reflects the limitations of the Y2H method, and is largely the result of protein fragmentation and the loss of physiological context (tissue and cell-state specificity of protein expression and subcellular localization). Thus, non-physiological interactions are possible and, in our case, more abundant than desirable. Conversely, fly orthologs of the previously identified LMBR1/LMBR1L interactors LCN1 [1,2] and ERAD and Wnt pathway components [9] were not identified in our screen. This is also not unexpected as our bait contained only the ICL3 region. Thus, LMBR1L’s interaction with LCN1 (which is mapped to its extracellular N terminus [1,2]) and the unmapped PPIs with ERAD/Wnt cascade components (which most likely involve one or more of the TMDs) were not expected in our screen.

Nonetheless, our Y2H screen offers a list of *potential* interactors that, in *Drosophila*, can be investigated based on temporal and spatial expression data, phenotypic analysis, and genetic interaction testing. We believe that a combination of these studies will allow us to home in on Lili’s true partners and specific biological and molecular function(s).

## Materials and methods

### Fly genetics

Fly constructs: pUASz1.0 vector was obtained from DGRC (DGRC Stock 1431). The BamHI site in its MCS was deleted and 6 bp were added between the XhoI and KpnI sites via mutagenesis PCR (Agilent PfuUltra II Hotstart PCR 2X Master Mix #600850). The *lili* CDS-linker-6xMyc was then subcloned 5’ to 3’ into the edited vector (referred to as pUASz) between XhoI and KpnI sites. Site directed mutagenesis was performed to generate new BamHI and BstEI sites in *lili* CDS (flanking ICL3) to generate the *pUASz-lili-6xMyc* backbone to subclone the ICL3 variants into. ICL3 variant sequences were synthesized as gene blocks from Genscript and were individually cloned 5’ to 3’ into the *pUASz-lili-6xMyc* backbone, between BamHI and BstEI, to generate corresponding *pUASz-lili variant X-6xMyc*.

All flies were grown at 25°C. Three *lili* null alleles were generated by CRISPR-Cas9 mediated deletion: *lili^Δ2^^–4^* null allele has a deletion of 517 bp from exon 2 to exon 4 of *lili* (S2 Fig) causing a frameshift leading to an early stop in exon 4 resulting in a gene product having 67 aa (first 38 aa are same as WT and the remaining are frameshifted, with a stop codon after aa 67). *lili^Δ4^* allele has an 8 bp deletion in exon 4, causing a frameshift and subsequent early truncation resulting in a gene product with 178 aa (first 153 aa are same as WT and the remaining are frameshifted, with a stop codon after aa 178) and *lili^Δ2^^–4long^* has two in-frame deletions from exon 2 to exon4, of a total of 529 bp, resulting in a gene product with 410 aa (gene product lacks aa 28 to aa 146 as compared to WT Lili, with an additional leucine and tyrosine after aa 27). Only *lili^Δ2^^–4^* was used in this study but all three allelic stocks are available for distribution on request. *lili^TG4^* is a gene trap generated as a Trojan GAL4 (TG4) tool [14]. The insertion of TG4 cassette lies in the first intron of *lili* such that the *lili* gene product is truncated while GAL4 is expressed under the native regulatory sequences of the locus (S2 Fig). *pUASz-Lili WT/variant X-6xMyc* containing transgenic flies were generated by site specific ϕC31 integrase mediated genomic insertion of corresponding sequences at the attP40 landing site on chromosome 2 (BestGene).

### *Drosophila* cell culture and protein extraction

Cell culture constructs: pAC-GAL4 was a gift from Liqun Luo (addgene plasmid 24344) [23]. The same UASz clones used to generate transgenic flies (see above) were used for Lili WT/variant expression in cell culture.

*Drosophila* S2-DRSC (S2) cells, purchased from the *Drosophila* Genomics Resource Center (DGRC stock 181), were grown at 25°-28 °C in M3 Medium (Sigma) supplemented with 10% heat-inactivated fetal bovine serum (SH30070.02, HyClone) and 50 units/mL penicillin G + 50 µg/mL streptomycin sulfate (Gibco). Cells were seeded at a density of 3x10^6^ cells per well in a 6-well culture dish. After 24 h, the cells were co-transfected with 1 µg of pUASz-Lili WT/variant X-6xMyc and 100 ng of pAc GAL4 using Mirus TransIT Insect (MIR 6100) reagent. 60h after transfection, the cells were scraped in media on ice, added to pre-chilled 2 ml microtubes and centrifuged at 500 rcf for 3 min at 4°C. The supernatant was discarded, and cell pellets were washed 3 times with ice cold PBS. The cells were lysed (pipette mixed and sonicated on ice for 3s) in ice cold 0.1% NP-40 lysis buffer consisting of 50 mM Tris HCl pH 8.0, 150 mM NaCl, Roche cOmplete™, Mini, EDTA-free Protease Inhibitor Cocktail #11836170001 (1 tablet/20 ml), Roche PhosSTOP Cat # 04906 837001 (1 tablet/20 ml). The tubes were then centrifuged at over 20,000 rcf for 8 min at 4°C, the supernatant was separated into fresh Eppendorf tubes and quantified using BCA assay (Thermo Scientific™ Pierce™ BCA Protein Assay Kits #PI23227).

### Amino acid sequence scrambling

Amino acids in Lili ICL3 were rearranged using the Sequence Scrambler at Peptide Nexus (https://peptidenexus.com/article/sequence-scrambler) to randomly generate an array of scrambled sequences. Structure of Lili with each generated scrambled ICL3 was predicted using Alphafold2 (https://neurosnap.ai/service/AlphaFold2) [24,25]. All scrambled peptide sequences reported in this paper (see S3 File for aa sequences) were selected after ensuring that the scrambled regions do not affect the overall transmembrane topology of the full-length protein, including TMD-loop boundaries and the length of TMDs/loops/termini. The scrambling approach retains overall charge of residues.

### Western blotting

30 µg of protein in Laemmli buffer (boiled for 5 min at 95°C, quick spun to collect condensate) was loaded into each well of a 10% SDS-PAGE gel, run at 200V for 30 min (Tris-Glycine buffer, 0.1% SDS pH 8.3) and transferred in Towbin buffer for 30 min at 15V using a Biorad semi-dry transfer system onto nitrocellulose membranes. Transfer efficiency was ensured using Ponceau S staining, followed by blocking with 5% non-fat dried milk in TBST (used to dilute primary and secondary antibodies also) for 1 h. The blots were then incubated with primary antibodies overnight, followed by three 5 min TBST washes, incubated with secondary antibodies for 2 h at room temperature and imaged and quantified using the LI-COR Odyssey CLx Imager. Antibodies: anti-Myc 1:1000 (Cell Signaling catalog #mAb 2278), anti-β-Actin 1:8000 (Cell Signaling catalog #mAb 4967), IRDye^®^ 680RD Goat anti-Rabbit IgG Secondary Antibody 1:20000 (product number 926–68071). Blots were analyzed and quantified using ImageJ and normalized to β-Actin levels. Full uncropped blots are provided in S4 File.

### Immunohistochemistry

Ovaries from 3–5-day old females were dissected in PBS and fixed in 3% paraformaldehyde/phospho-lysine buffer for 30 min, followed by three washes each of 1x PBS and 1x PBS-Triton-X-100 (0.3% Triton-X-100, PBST). Tissue was blocked for 30 min in 5% normal goat serum/PBST and incubated with primary antibodies in fresh blocking solution for 24 h. Tissue was washed three times each in PBST and blocking solution and incubated with secondary antibodies in fresh blocking solution overnight. Tissue was then washed three times each in PBST and PBS before mounting. Primary antibodies were from the Developmental Studies Hybridoma Bank 1:50 rat α-DE-Cad (clone DCAD2, deposited by T. Uemura), Cell Signaling [1:200 rabbit α-Myc (catalog #mAb 2278). Cy3-, and Cy5-conjugated goat α-rat or rabbit secondary antibodies were used at 1:400 (Jackson ImmunoResearch). The tissue was mounted in Vectashield (Vector Labs).

### Image acquisition and processing

Confocal stacks were recorded in Leica LASX software using a Leica DM5500Q microscope with SPEII confocal head. Images were processed in LASX; Quantifications was done using ImageJ. Signal intensity from every triskelion (minimal repeating unit per image) per image was measured, corresponding background noise subtracted and averaged per image, ensuring signal from equal volume was being compared. Images were assembled using Adobe Illustrator.

### Yeast 2-Hybrid screen (Hybrigenics)

The bait fragment consists of Lili amino acids 219–352 (134 aa) which spans the predicted cytosolic ICL3, with a few flanking TM residues on either side (Fig 2A) to ensure that its predicted properties (folding and structure) closely reflect that of the WT ICL3, with or without GAL4 DNA binding domain (DBD) fusion (predictions performed using AlphaFold2). The screen (ULTImate Y2H™) was performed by Hybrigenics Services, S.A.S. (www.hybrigenics-services.com). The coding sequence for aa219-aa352 of *Drosophila melanogaster Lili* (NM_143033.3) was PCR-amplified and cloned into pB66 as a C-terminal fusion to the GAL4 DNA binding domain (DBD). Sequencing of the vector was done to ensure that the insert was in frame with the GAL4 DBD. pB66 derives from the original pAS2ΔΔ vector [26]. Lili ICL3 was used as bait to screen against a *Drosophila melanogaster* whole embryo stage 0–12 h and 12–24 h cDNA library (DME_RP2) constructed in pP6 and containing 10 million fragments in yeast. pP6 is derived from the original pGADGH [27] plasmid. Using a mating approach with CG1945 (mata) and YHGX13 (Y187 ade2–101::loxP-kanMX-loxP, matα) yeast strains, one hundred and five million clones (10X library complexity) were screened. On DO-3, 275 His+ colonies were selected. Prey fragments from these His+ clones were PCR amplified and sequenced at their 5′ and 3′ regions. A fully automated procedure was used to map the sequencing results to the respective interaction partners in the GenBank database (National Center for Biotechnology Information). A confidence score—Predicted Biological Score (PBS)—was attributed to each interaction to assess the interaction reliability. This score represents the probability of an interaction to be non-specific: it is an *e*-value, primarily based on the comparison between the number of independent prey fragments found for an interaction and the chance of finding them at random (background noise). Several thresholds have been arbitrarily defined to rank the results into 4 categories from A (the highest confidence rank) to D, with technical artifacts/non-specific interactors categorized into 2 categories E and F (see Supplemental File 1 for additional details).

The prey fragments of Mad (aa 72-436; NM_001273063.1 transcript variant B), Sara (aa 717-1046, NM_079990.3), Sesn (aa 262-497; NM_137977.4 transcript variant A) and EDTP (aa 155-227; NM_206151.2 transcript variant C) were derived from interaction clones obtained from the screen; they contain the minimum length of amino acid sequence shared by all prey fragments matching the same reference protein. These fragments were ensured to be in frame with the GAL4 activation domain (AD) in pP6.

### Yeast 2-Hybrid assays

Yeast 2-Hybrid constructs: The ICL3 bait used in the screen, pB66-Lili ICL3, was designed by SJN and generated by Hybrigenics. All other baits were generated by MVR by deletion/mutagenesis PCR of pB66-Lili ICL3.

Bait and prey constructs were co-transformed into yeast diploid cells (auxotrophic for leu, trp and his, obtained from mating of CG1945 mat-a and YHGX13 mat-α, from Hybrigenics). These assays are based on the HIS3 reporter gene (growth assay on media lacking histidine). As negative controls, the empty pB66 (pB66φ) and baits were tested in the presence of empty prey vector pP6 (pP6φ) and all prey plasmids were tested with pB66φ. For each tested interaction, multiple dilutions of cells (culture normalized at 3 × 10^7^ cells/ml; undiluted, 10^-1^, 10^-2^, 10^-3^) transformed with both bait and prey constructs were spotted on selective media. The DO-2 selective medium (-Trp, -Leu) was used as a growth control to ensure the presence of both the bait and prey plasmids. Multiple dilutions were spotted on selective medium DO-3 (-Trp, -Leu, -His). Different concentrations of 3-AT, an inhibitor of the HIS3 gene product, were added to the DO-3 plates to increase stringency and overcome possible autoactivation by bait or prey. 3-AT was also used to assess the strength of bait-prey interactions as growth on higher concentrations of 3-AT suggested stronger PPI strength. The following 3-AT concentrations were tested: 0.2, 0.5, 1,2 and 5 mM. All plates were incubated at 30°C for 3–5 days and scored. Reproducibility has been ensured by independently repeating all bait-prey combinations in Y2H assays at least twice, with multiple technical replicates.

## Supporting information

S1 Fig Predicted structure of Lili and its sequence conservation. A) Predicted structure of Lili (AlphaFold), visualized using UCSF Chimera 1.15, with polar residues in blue and hydrophobic residues in red. B) Sequence alignment of Lili and its homologs across multiple metazoans, residues highlighted in black are identical and gray are similar. IC loops are labeled and color coded same as in the predicted structure in Fig 1A. Lili shares over 40% sequence identity with reported LMBR1/-like proteins (LMBR1 42.4%, LMBR1L 44.6% in zebrafish; LMBR1 43%, LMBR1L 45% in Xenopus; LMBR1 43.6%, LMBR1L 45.9% in mouse; LMBR1 43.6%, LMBR1L 45.4% in human). TMD: transmembrane domain.
(TIF)

S2 Fig Illustration of the Lili locus and *lili* null alleles used in the phenotypic rescue assay. A schematic representation of the *lili* genomic locus as per Flybase (June 25, 2024). The orange boxes denote the gene’s ORFs. *lili*^*Δ2*^^*–4*^ was generated by CRISPR-Cas9 mediated deletion of 517 bp spanning from exon 2 to exon 4. *Mi{Trojan-GAL4.0}lili^MI10456-TG4.0^* (*lili*^*TG4*^) is a gene trap null allele generated by insertion of the Trojan GAL4 (TG4) cassette in the first intron of *lili* [14]. The TG4 cassette consists of a splice acceptor, T2A peptide, GAL4 coding sequence and an Hsp70 transcription termination signal.
(TIF)

S3 Fig Expression of Myc tagged Lili WT and variants *in vitro and in vivo.* A) Myc tagged Lili WT and ICL3 variants that failed to rescue larval lethality were transiently expressed in *Drosophila* S2 cells and levels were assessed by immunoblotting. Empty vector (EV; pUASz vector) was used as a control. Variant with ICL3 substituted with ICL2 has a different mobility compared to WT owing to its lower molecular weight (Lili WT-6xMyc: 72 kDa, Lili-ICL2 substitution: 60 kDa). B) Myc tagged Lili WT and ICL3 variants that successfully rescued larval lethality were transiently expressed in *Drosophila* S2 cells and levels were assessed by immunoblotting. Empty vector (EV; pUASz vector) was used as a control. Variants can be robustly expressed in S2 cells. Variant with ICL3 substituted with LMBR1/LMBR1L ICL3 have different mobilities compared to WT owing to its lower molecular weight (Lili LMBR1 ICL3 substitution: 67 kDa, Lili LMBR1L ICL3 substitution: 67 kDa). Band intensities are reported after normalization to loading control β-Actin. C) Comparison of expression levels of all Lili ICL3 variants as compared to Lili WT transgene expression in S2 cells and ovaries. Though the expression levels vary among variants both *in vivo* and *in vitro*, all are expressed robustly and would be expected to rescue if functional. D) When overexpressed in the *lili* pattern (*lili*^*TG4*^* > UASz-Lili WT/variant-6xMyc*), the Myc tagged WT protein can be easily detected in the nurse cells of stage 9/10 egg chambers. Variants that fail to rescue larval lethality in phenotypic assays show expression patterns indistinguishable from that of exogenous WT Lili-Myc. Quantification was by ImageJ, averaging signal from all triskelions (white box) per image.
(TIF)

S4 Fig Validation of Lili ICL3 PPI with Mad, Sara, Sesn and EDTP by Hybrigenics. A) Tabulated raw data from Y2H assays performed by Hybrigenics, verifying Lili ICL3-Mad/Sara/Sesn/EDTP interactions obtained from the screen, seen by growth on selection media lacking histidine and -his + 3-AT. B) Colony growth on selection media, scored 5 days after plating to generate the data table in A). Growth on DO-3 was titrated with increasing concentrations of 3-AT for stringency. + : > 10 colonies, + /-: < 10 colonies, -: no colonies on selective media.
(TIF)

S1 Table Observations supporting summary data presented in Fig 1C. (XLSX)

S1 File Hybrigenics Y2H screen results and Gene Ontology analysis. (XLSX)

S2 File Tabulated data from Y2H assays testing for Lili ICL3 PPIs with Mad, Sara, Sesn and EDTP. (XLSX)

S3 File Amino acid sequence information of all Lili ICL3 variants and Y2H baits. (XLSX)

S4 File Uncropped western blots related to S3 Fig. (PDF)
