# Supplementary figures and images for: The third intracellular loop of Drosophila Lilipod is required for protein function in vivo and can mediate protein-protein interactions in vitro
Source: PLoS One. 2025 Jun 4;20(6):e0325326. doi: 10.1371/journal.pone.0325326 (PMC12136406; doi:10.1371/journal.pone.0325326)

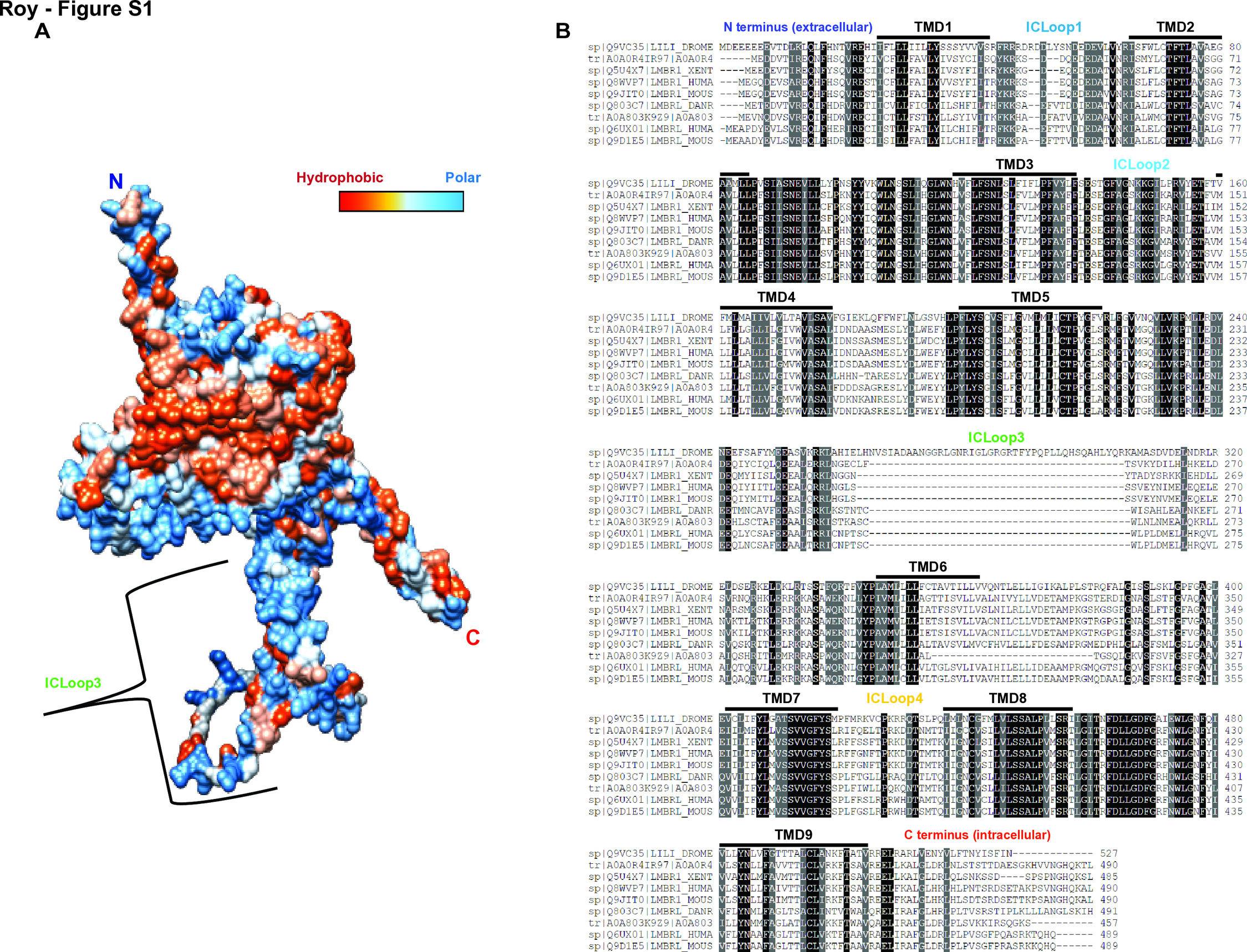

Supplement: S1 Fig — A) Predicted structure of Lili (AlphaFold), visualized using UCSF Chimera 1.15, with polar residues in blue and hydrophobic residues in red. B) Sequence alignment of Lili and its homologs across multiple metazoans, residues highlighted in black are identical and gray are similar. IC loops are labeled and color coded same as in the predicted structure in Fig 1A. Lili shares over 40% sequence identity with reported LMBR1/-like proteins (LMBR1 42.4%, LMBR1L 44.6% in zebrafish; LMBR1 43%, LMBR1L 45% in Xenopus; LMBR1 43.6%, LMBR1L 45.9% in mouse; LMBR1 43.6%, LMBR1L 45.4% in human). TMD: transmembrane domain. (TIF) [file pone.0325326.s001.tif]

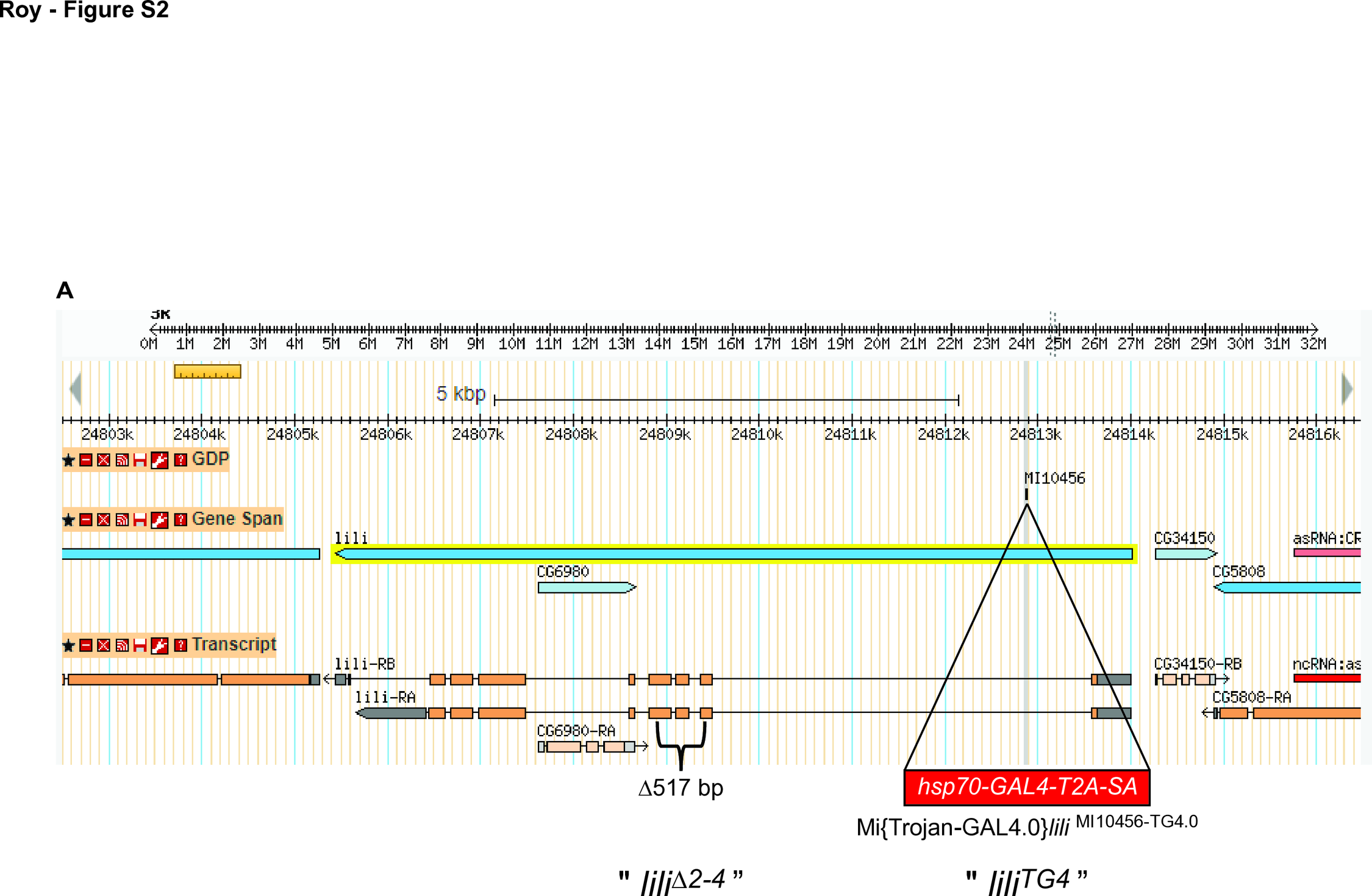

Supplement: S2 Fig — A schematic representation of the lili genomic locus as per Flybase (June 25, 2024). The orange boxes denote the gene’s ORFs. liliΔ2–4 was generated by CRISPR-Cas9 mediated deletion of 517 bp spanning from exon 2 to exon 4. Mi{Trojan-GAL4.0}liliMI10456-TG4.0 (liliTG4) is a gene trap null allele generated by insertion of the Trojan GAL4 (TG4) cassette in the first intron of lili [14]. The TG4 cassette consists of a splice acceptor, T2A peptide, GAL4 coding sequence and an Hsp70 transcription termination signal. (TIF) [file pone.0325326.s002.tif]

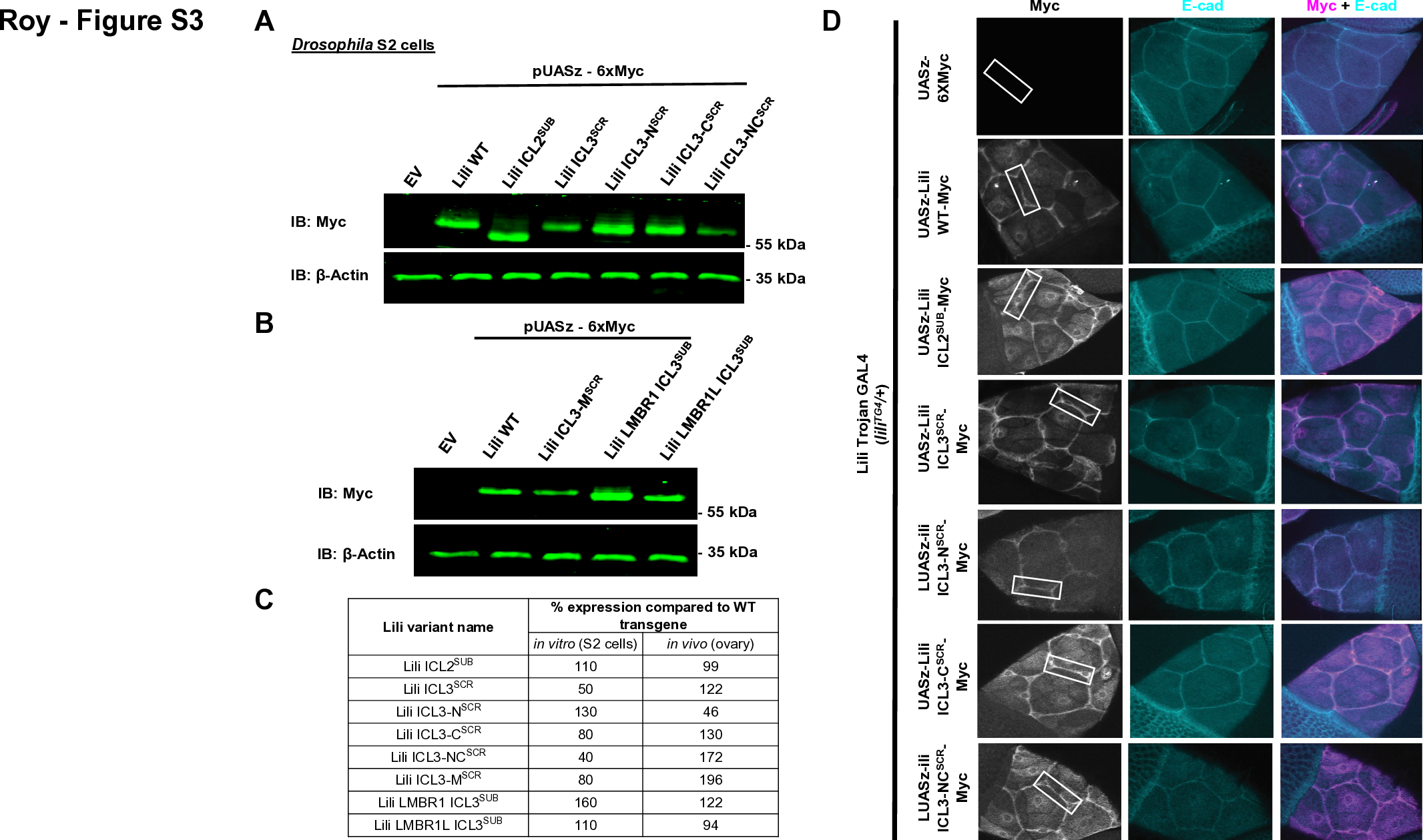

Supplement: S3 Fig — A) Myc tagged Lili WT and ICL3 variants that failed to rescue larval lethality were transiently expressed in Drosophila S2 cells and levels were assessed by immunoblotting. Empty vector (EV; pUASz vector) was used as a control. Variant with ICL3 substituted with ICL2 has a different mobility compared to WT owing to its lower molecular weight (Lili WT-6xMyc: 72 kDa, Lili-ICL2 substitution: 60 kDa). B) Myc tagged Lili WT and ICL3 variants that successfully rescued larval lethality were transiently expressed in Drosophila S2 cells and levels were assessed by immunoblotting. Empty vector (EV; pUASz vector) was used as a control. Variants can be robustly expressed in S2 cells. Variant with ICL3 substituted with LMBR1/LMBR1L ICL3 have different mobilities compared to WT owing to its lower molecular weight (Lili LMBR1 ICL3 substitution: 67 kDa, Lili LMBR1L ICL3 substitution: 67 kDa). Band intensities are reported after normalization to loading control β-Actin. C) Comparison of expression levels of all Lili ICL3 variants as compared to Lili WT transgene expression in S2 cells and ovaries. Though the expression levels vary among variants both in vivo and in vitro, all are expressed robustly and would be expected to rescue if functional. D) When overexpressed in the lili pattern (liliTG4 > UASz-Lili WT/variant-6xMyc), the Myc tagged WT protein can be easily detected in the nurse cells of stage 9/10 egg chambers. Variants that fail to rescue larval lethality in phenotypic assays show expression patterns indistinguishable from that of exogenous WT Lili-Myc. Quantification was by ImageJ, averaging signal from all triskelions (white box) per image. (TIF) [file pone.0325326.s003.tif]

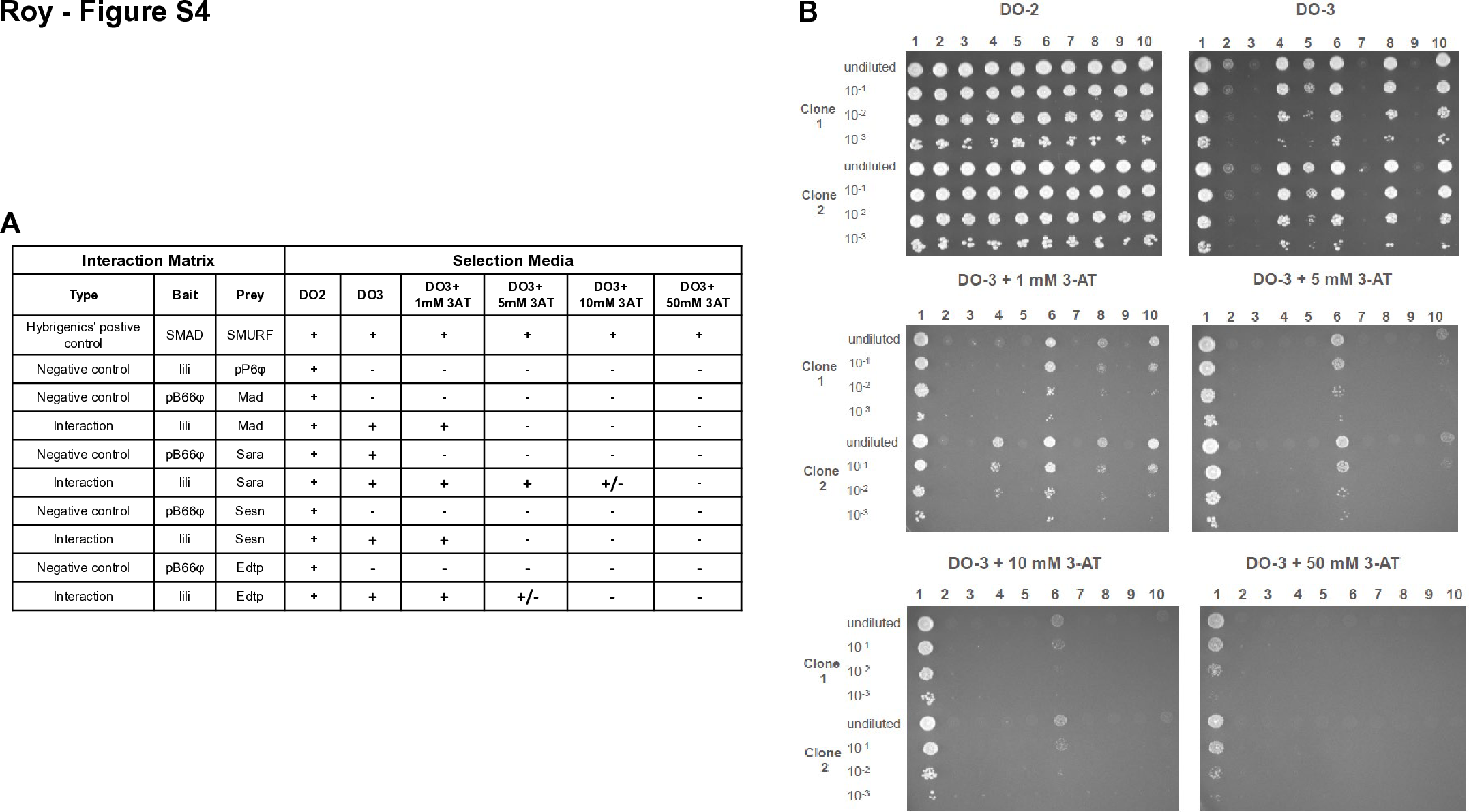

Supplement: S4 Fig — A) Tabulated raw data from Y2H assays performed by Hybrigenics, verifying Lili ICL3-Mad/Sara/Sesn/EDTP interactions obtained from the screen, seen by growth on selection media lacking histidine and -his + 3-AT. B) Colony growth on selection media, scored 5 days after plating to generate the data table in A). Growth on DO-3 was titrated with increasing concentrations of 3-AT for stringency. + : > 10 colonies, + /-: < 10 colonies, -: no colonies on selective media. (TIF) [file pone.0325326.s004.tif]

Roy - Full Blots (Figure S3)

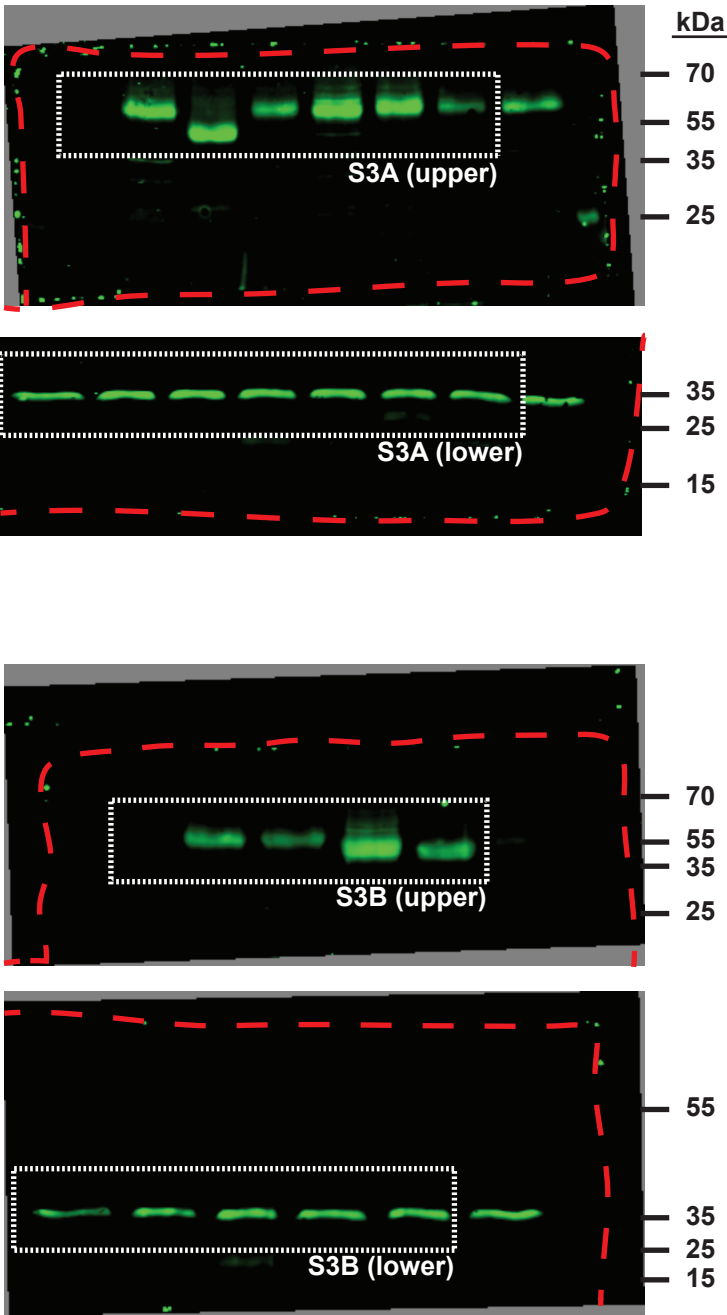

Supplement: S4 File — (PDF) [file pone.0325326.s009.pdf]
